# Highly Stretchable Bacterial Cellulose Produced by *Komagataeibacter hansenii* SI1

**DOI:** 10.3390/polym13244455

**Published:** 2021-12-19

**Authors:** Izabela Cielecka, Małgorzata Ryngajłło, Waldemar Maniukiewicz, Stanisław Bielecki

**Affiliations:** 1 Institute of Molecular and Industrial Biotechnology, Lodz University of Technology, 90-573 Łódź, Poland; malgorzata.ryngajllo@p.lodz.pl (M.R.); stanislaw.bielecki@p.lodz.pl (S.B.); 2 Institute of General and Ecological Chemistry, Lodz University of Technology, 90-924 Łódź, Poland; waldemar.maniukiewicz@p.lodz.pl

**Keywords:** *Komagataeibacter*, stretchable bacterial cellulose, enhanced strain, vitamin C

## Abstract

A new strain of bacteria producing cellulose was isolated from Kombucha and identified as *Komagataeibacter hansenii*, named SI1. In static conditions, the strain synthesises bacterial nanocellulose with an improved ability to stretch. In this study, utilisation of various carbon and nitrogen sources and the impact of initial pH was assessed in terms of bacterial nanocellulose yield and properties. *K. hansenii* SI1 produces cellulose efficiently in glycerol medium at pH 5.0–6.0 with a yield of 3.20–3.60 g/L. Glucose medium led to the synthesis of membrane characterised by a strain of 77%, which is a higher value than in the case of another *Komagataeibacter* species. Supplementation of medium with vitamin C results in an enhanced porosity and improves the ability of bacterial nanocellulose to stretch (up to 123%). The properties of modified membranes were studied by scanning electron microscopy, Fourier transform infrared spectroscopy, X-ray diffraction and mechanical tests. The results show that bacterial nanocellulose produced in SH medium and vitamin C-supplemented medium has unique properties (porosity, tensile strength and strain) without changing the chemical composition of cellulose. The method of production BNC with altered properties was the issue of Polish patent application no. P.431265.

## 1. Introduction

Biopolymers produced by bacteria, e.g., polyamides, polyesters, polysaccharides and extracellular proteins, are the part of the bacteria inherent physiology. They outperform properties of polymers extracted from natural origin because microbial biopolymers can be modified to specific application by biotechnology tools [1]. Bacterial nanocellulose (BNC) is an increasingly used natural polymer in constructing new smart biomaterials that can be applied in many fields [2]. It is produced in nanostructured membranes by many bacterial strains, such as *Komagataeibacter*, *Agrobacterium*, *Sarcina* or *Rhizobium* [3]. The most studied cellulose producers are the *Komagataeibacter* species, synthesising BNC in a pellicle at the air/liquid interface [4]. The chemical structure of BNC is identical to that of the plant cellulose, but it does not contain impurities such as hemicelluloses, lignin or dyes. Bacteria can produce cellulose from different carbon sources, including monosaccharides (glucose, fructose, galactose), disaccharides (sucrose, maltose) and sugar alcohols (glycerol, mannitol) [5]. Much research has recently focused on screening new strains producing BNC efficiently or with unique properties. *Komagataeibacter* strain could be isolated from Kombucha [6], vinegar [7,8], rotten green grapes [9], coconut milk [10] or fruits [11].

The structure of BNC consists of nanofibrils assembled into ultrathin fibres with a width of approximately 8 nm and next into ribbons of 70–150 nm width. Those ribbons are organised into the three-dimensional network [12]. The structure of BNC determines unique properties of this biopolymer, such as mechanical stability, high porosity, high water holding capacity (up to 99%) and crystallinity over 74% [13]. Moreover, the biocompatibility of BNC is well documented, which makes it a suitable material for biomedical and tissue engineering applications [14,15]. Physicochemical properties of bacterial cellulose has aroused the interest of a wide range of industries, where it can be useful as a part of biosensors [16], food and cosmetics stabilisers [17], packaging [18] and drug or enzymes delivery matrix [19,20]. The scope of BNC applications is highly dependent on biomaterial structure, defined properties and production costs. In the reported research, two main trends can be observed. The first of them regards to the economical aspects of BNC production. There are sought new, efficient strains, the culture conditions are optimised, and cheaper carbon sources are tested [21]. The second approach is attributed to in situ and ex situ methods of BNC properties modification. Those efforts are focused on the achievement of biomaterial with desirable features. Specific parameters of BNC membrane depend on fibres organisation in the three-dimensional structure, amount of branching points, the width of fibres, crystallinity and water content [22]. Those properties can be influenced by biological, engineering and material sciences approaches. Physico-chemical parameters of BNC can be affected by the producing strain, culture conditions, culture method and a wide range of additives. Cellulose produced in static conditions is formed as a flat pellicle with a dense three-dimensional structure. In contrast, agitated fermentation generates BNC in a spherical shape and is characterized by an enhanced porosity and looser arrangement of fibres in the structure [23]. On the other hand, it is known that different strains can produce cellulose with various crystallinity [24]. *Gluconacetobacter xylinus* ATCC 10,245 is able to produce BNC with crystallinity at 88% [25], while the crystallinity of cellulose synthesised by *G. xylinus* PTCC 1734 is only 63% [26]. In our previous study, the impact of culture conditions (such as culture time, glucose concentration, pH of culture medium and air-flow ratio) on mechanical strength has been shown [27].

In the presented research, a new cellulose producing strain was isolated and identified as *Komagataeibacter hansenii*, named SI1. This strain was microbiologically characterised, and kinetic growth, BNC structure, chemical composition, and BNC accumulation have been studied. Next, various culture conditions (carbon source, nitrogen source, pH, culture additives) were investigated for BNC production by *K. hansenii* SI1. Bacterial cellulose produced in the glucose medium exhibited unique mechanical properties and high porosity. Which were further improved by supplementation of vitamin C. Afterwards, the metabolism of *K. hansenii* SI1 growing in both control conditions and vitamin C-supplemented medium was assessed and compared during a 7-day culture. Next, modified cellulose membranes were analysed in the context of structure arrangement by scanning electron microscopy, chemical composition, mechanical properties, crystallinity and crystallite size.

## 2. Materials and Methods

### 2.1. Isolation and Identification of Microorganism

The bacterial cellulose (BNC) producing bacteria were isolated from commercial Kombucha beverages available in the Polish market. A 1 mL sample was inoculated into 10 mL of the Schramm–Hestrin (SH) medium consisting of 20 g/L glucose, 5 g/L yeast extract, 5 g/L peptone, 2.7 g/L Na_2_HPO_4_, 1.15 g/L citric acid and 0.5 g/L MgSO_4_ with pH 5.7 adjusted with 0.1 M acetic acid. The culture was incubated at 30 °C for three days in static conditions until a pellicle on the culture surface appeared. Afterwards, 0.1 mL of those cultures were spread in a solid SH-agar (2% agar) medium containing cyclohexamide (0.1 g/L) and incubated at 30 °C for three days. Potential cellulose-producing bacteria were separately transferred into 5 mL of SH medium and incubated at 30 °C for three days. Next, from each culture with a pellicle, 0.1 mL was spread on an SH-agar medium and incubated. The procedure was repeated until single colonies were obtained. After three days, pure colonies were cultivated in 5 mL of SH medium and transferred into 100 mL of fresh SH medium. Cultures were incubated at 30 °C for seven days, and the microorganism for genetic identification was chosen based on cellulose characteristics. The BNC producer was preserved under freezing (−80 °C) using 20% glycerol as a cell cryoprotectant.

The genomic DNA of Kombucha was isolated as described by Ryngajłło et al. [28]. Genome sequencing was performed by BioNanoPark Łódź, Poland. Briefly, NGS libraries were prepared using NEBNext DNA Library Preparation Kit (New England Biolabs, Ipswich, MA, USA). Genome sequencing was performed using the Illumina MiSeq platform (Illumina, San Diego, CA, USA) in 2 × 250 bp paired-end reads mode. The sequencing reads were assembled de novo using SPAdes (v. 3.6.2, [29]). The genome sequence of Kombucha strain has been deposited into the NCBI database under the BioProject number: PRJNA751727.

Comparisons of genomic similarity are currently considered a standard for species classification. In particular, the in silico comparisons involving whole genome sequence allows for obtaining exact results. One of the most accurate genome-wide similarity statistics is average nucleotide identity (ANI) [30]. ANI-based phylogenetics of the newly isolated cellulose producers is now possible due to the availability of the type strain genome sequences for the species of the *Komagataeibacter* genus [31]. ANI analysis was performed using PYANI (v. 0.2.9; [32]) python program employing BLAST+ program [33]. The UPGM tree based on ANI-1 values was calculated using the phangorn R package [34]. Genome sequences of 16 type *Komagataeibacter* strains and *Glouconacetobacter entanii* LTH 4560 strain were downloaded from NCBI [31].

### 2.2. Culture Conditions

#### 2.2.1. Inoculum Preparation

Bacteria from frozen stock were activated by spreading on SH-agar plate and incubation at 30 °C for three days. Next, a single colony was transferred into 5 mL of SH medium and incubated at 30 °C for three days. Then, cultures were transferred into 100 mL of SH medium and incubated at 30 °C. After three days, culture was used as an inoculum.

#### 2.2.2. Culture Medium

In this study, Schramm–Hestrin medium, containing 20 g/L glucose (POCh, Gliwice, Poland), 5 g/L yeast extract (BTL, Łódź, Poland), 5 g/L bacterial peptone (BTL, Łódź, Poland), 2.7 g/L sodium phosphate dibasic (Chempur, Piekary Śląskie, Poland), 1.15 g/L citric acid (Chempur, Piekary Śląskie, Poland) and 0.5 g/L magnesium sulfate (Chempur, Piekary Śląskie, Poland), was used as the basal medium. The initial pH of the SH medium was adjusted to 5.7 using 0.1 M acetic acid.

### 2.3. Primary Characterisation of Bacterial Cellulose Production by the Komagataeibacter hansenii SI1 Strain

In our study, we assessed the kinetics of BNC production in standard conditions (SH medium, 30 °C, seven days) and BNC properties. Colonies were observed by using a microscope after four days. A time-course experiment determined growth rate, glucose consumption, BNC accumulation, and pH for seven days. The structure was visualised by a macro-photograph and scanning electron microscopy (SEM). Fibres width was determined based on SEM images as described in Section 2.7.2.

### 2.4. Time Course of BNC Biosynthesis

The metabolism of *K. hansenii* SI1 was studied for SH medium (primary characterisation) and SH medium supplemented with ascorbic acid at 0.5% and 1.0% concentration. The *K. hansenii* SI1 strain was cultivated in 5 mL of medium using 10 mL test tubes. Each replicate was inoculated with a single colony and incubated at 30 °C for seven days. The kinetic study was carried out on each day. The BNC yield was calculated as the dry weight of purified membranes per 1 L of culture medium. Bacterial growth as CFU was evaluated using the serial dilution method after degradation of cellulose membrane with cellulase (100 µL/culture, dilution of 5:3 in SH medium, Ultraflo Max, Novozymes, Kalundborg, Denmark). After serial dilution, 100 µL of the cell suspension was spread on a solid SH medium. The colony-forming units were counted after three days of incubation and expressed as a log(CFU). According to the manufacturer’s protocol, the glucose concentration was assessed using a GLUCOSE test (BioMaxima, Lublin, Poland). The concentration of residual ascorbic acid was determined using a K-ASCO assay kit (Megazyme, Bray, Ireland). Each test was performed at least in three replicates.

### 2.5. Impact of Culture Conditions on Bacterial Cellulose Yield

In this study, the impact of carbon and nitrogen source was evaluated. Thus, glucose was substituted with one of the following carbon sources: fructose (Chempur, Piekary Śląskie, Poland), galactose (Chempur, Piekary Śląskie, Poland), glycerol (Chempur, Piekary Śląskie, Poland), lactose (Chempur, Piekary Śląskie, Poland), maltose (Chempur, Piekary Śląskie, Poland), mannitol (Chempur, Piekary Śląskie, Poland) and sucrose (Chempur, Piekary Śląskie, Poland). The concentration of carbon source in each variant was always 20 g/L. Standard nitrogen sources (yeast extract and peptone; BTL, Łódź, Poland) were substituted with another, added individually to SH medium with the concentration of 5 g/L, namely ammonium sulphate (Chempur, Piekary Śląskie, Poland), sodium nitrate (Chempur, Piekary Śląskie, Poland), urea (Chempur, Piekary Śląskie, Poland), corn steep liquor (Merck, Darmstadt, Germany), peptone (BTL, Łódź, Poland) or yeast extract (BTL, Łódź, Poland). The influence of BNC enhancers was studied for ethanol (1%) (Chempur, Piekary Śląskie, Poland), lactic acid (0.6%) (Merck KGaA, Darmstadt, Germany) and vitamin C (0.5% and 1.0%) (Stanlab, Lublin, Poland). The control was a culture grown in the SH medium.

Each culture was prepared in cuboid bioreactors filled with 200 mL of the modified SH medium. The medium was inoculated with 5% inoculum and cultivated for seven days at 30 °C. Afterwards, membranes were collected and purified according to Cielecka et al. [35]. The BNC membranes were then dried at 90 °C in a gel drier (Bio-Rad Laboratories, Hercules, CA, USA) until a constant weight was achieved [36]. The biosynthesis yield was expressed as the dry weight of membrane obtained from 1 L of culture medium. The samples were cultured in triplicate for each variant of the medium.

### 2.6. Evaluation of the Impact of Ascorbic Acid on Bacterial Cellulose Production and Properties

The influence of vitamin C on BNC biosynthesis was assessed in a time-course experiment described in the time course experiment section. The experiment was performed using two concentrations of vitamin C, namely 0.5% and 1.0%. Before adding to the culture medium, vitamin C was dissolved in SH medium (10 g/L) and sterilised using a syringe filter (0.22 µm). Next, an appropriate volume was added to each medium sample (5 mL) placed in the test tube. The supplemented medium was inoculated with a single colony. Cultures were incubated at 30 °C for seven days. Moreover, we evaluated the arrangement of fibres in the three-dimensional structure, chemical composition by FTIR analysis, crystallinity and average crystallite size and mechanical properties.

### 2.7. Analytical Methods

#### 2.7.1. Mechanical Strength

The membranes were examined for tensile strength using a universal testing machine (Zwick/Roell Z1.0, Ulm, Germany), according to the method described by Cielecka et al. [35], with slight changes. The BNC was pressed before the tensile tests until 1 mm thickness was achieved to remove any excess water attached to membranes. Next, membranes were carefully cut into rectangular samples (20 mm × 45 mm) with a scalpel. The thickness of a strip was measured using a digital calliper. The samples were placed between two clamps (the gauge length was 15 mm) and subjected to deformation at a rate of 10 mm/min, while the pre-load was 0.1 N. The maximum stress and elongation at break were estimated using TestXpert@II software. Stress (MPa) was calculated as F/A, where F is the loading force expressed in Newtons (N), and A is the cross-section area of a sample. Strain (%) was calculated as ΔL/L_0_ × 100%, where L_0_ is the initial length, and ΔL is the exerted extension from starting point. Values for Young’s modulus (YM) under tension were calculated from the stress/strain relationship in the first linear region of the graph. The measurements were performed in at least nine replicates.

#### 2.7.2. Scanning Electron Microscopy

Before testing, the samples were freeze-dried and then sputter-coated with a gold layer. A FEI QUANTA 250 FEG microscope (Thermo Fischer Scientific, Waltham, MA, USA) was used to visualise BNC structure with scanning parameters: HV = 2 kV and magnification × 40,000, for each sample. Fibre thickness was evaluated using Makroaufmassprogramm software (open source software by Jens Rüdig, http://ruedig.de/tmp/messprogramm.html, accessed on 3 April 2017).

#### 2.7.3. Fourier Transform Infrared Spectrometry in Attenuated Total Reflectance Mode Analysis

Before testing, samples were freeze-dried in an ALPHA 1–2/LD freeze dryer (Martin Christ GmbH, Osterode am Harz, Germany). Chemical analysis of variations in the structure of BNC produced under different conditions was performed by FT-IR with attenuated total reflectance mode (ATR). The spectra were recorded at a resolution of 8 cm^−1^, in the range of 4000 to 650 cm^−1^ using a Nicolet 6700 FT-IR (Thermo Fischer Scientific, Waltham, MA, USA). For each sample, 200 scans were taken.

#### 2.7.4. X-ray Diffractometry

Room temperature powder X-ray diffraction patterns were collected using a PANalytical X’Pert Pro MPD diffractometer (Malvern Panalytical Ltd., Malvern, UK) in the Bragg–Brentano reflection geometry and the graphite monochromated Cu-Kα radiation. The PANalytical X’Celerator detector was used. All data were collected in the 2θ range 5–60° with a step of 0.0167° and an exposure per step of 30 s. The samples were spun during data collection to minimise preferred orientation effects. A PANalytical X’Celerator detector based on the Real-Time Multiple Strip technology and simultaneously measuring intensities in the 2θ range of 2.122° was used. The WAXFIT program was used to resolve the X-ray diffraction patterns [37]. The crystallinity index was calculated as the ratio of the integral intensity under all crystalline peaks to the sum of integral intensity under the crystalline peaks and amorphous halo [38]. The initial positions of crystalline peaks were assumed in accordance with the literature data [39]. According to the hybrid optimisation procedure, each diffraction curve was analysed by creating a theoretical function best fitted to the experimental curve, which combines a genetic algorithm with a modified Rosenbrock optimisation method. A linear combination of the Gauss and Cauchy profiles were used to construct the theoretical function approximating crystalline peaks and an amorphous halo. As a result of fitting, all the parameters of the component functions were determined.

The average crystallite size (ACS) was calculated according to the Scherrer equation [40]. The broadening of diffraction peaks due to crystallite size can be expressed as:

Crystallite size (average) = K λ/(Bscos θ) (1)

where: Bs is broadening due solely to crystallite size, K is a constant, the value of which depends on the particle shape (taken as 0.9 in this case), θ is the Bragg’s angle, and λ is the wavelength of the incident X-ray beam.

## 3. Results and Discussion

### 3.1. Strain Classification

**Figure 1:**
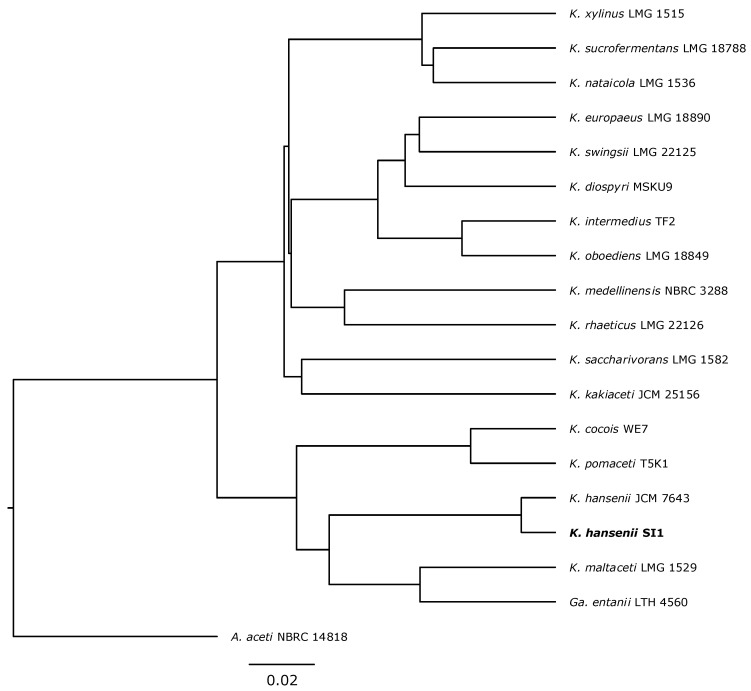
Phylogenetic relationship of the newly isolated Kombucha strain and type *Komagataeibacter* strains. The phylogenetic tree was constructed on ANI-1 values calculated based on whole-genome alignment. The *A. aceti* NBRC 14,818 was used as an outgroup. The tree was drawn in the FigTree program (v. 1.4.4). The scale bar represents the sequence divergence.

To analyse the phylogenetic relationship of the newly isolated strain with *Komagataeibacter* species, its genome was sequenced (manuscript describing the genome is in preparation). The whole-genome sequence comparison with type *Komagataeibacter* strains revealed that the strain isolated from Kombucha clustered with *K. hansenii* JCM 7643 strain (Figure 1). Based on these results, the new strain was classified as *K. hansenii* SI1.

### 3.2. Characterisation of Strain K. hansenii SI1, BNC Properties and Kinetics of Biosynthesis in Standard Conditions

**Figure 2:**
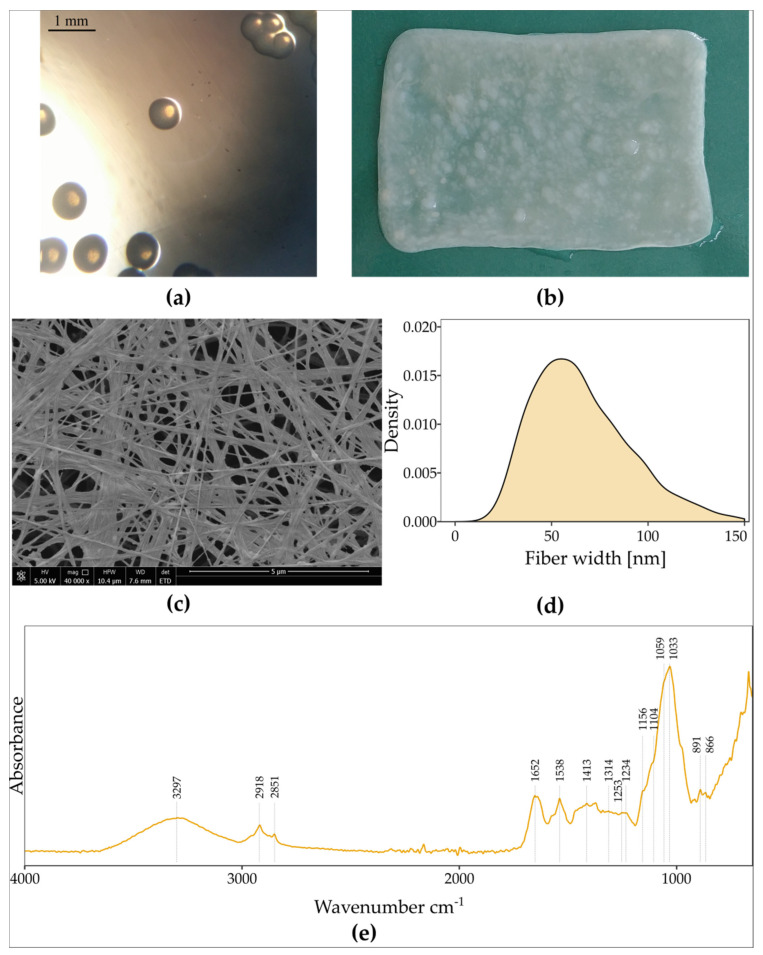
(**a**) Three-days colonies of *K. hansenii* SI1, (**b**) BNC membrane produced in SH medium, (**c**) scanning electron microscope image of BNC membrane produced in SH medium; the image was recorded at a magnification of 40,000× (bar—5 µm), (**d**) the probability density function of fibre width in BNC produced in SH medium and (**e**) FTIR-ATR spectrum of BNC produced in SH medium.

This study isolated a new cellulose-producing strain from a commercial Kombucha beverage. The isolate produced smooth, pale yellow colonies with a circular shape and a diameter of approximately 0.72 mm (Figure 2a). Bacteria were characterised as Gram-negative, aerobic microorganisms which did not form spores. Bacteria were rod-shaped, occurring singly or in short chains. We found that it metabolised carbohydrates and sugar alcohols and grew in the presence of ethanol and lactic acid. In liquid media, bacteria produced a pellicle at the surface of the medium (Figure 2b), which has a three-dimensional structure (Figure 2c) consisting of fibres with a diameter of 10–150 nm (Figure 2d).

**Figure 3:**
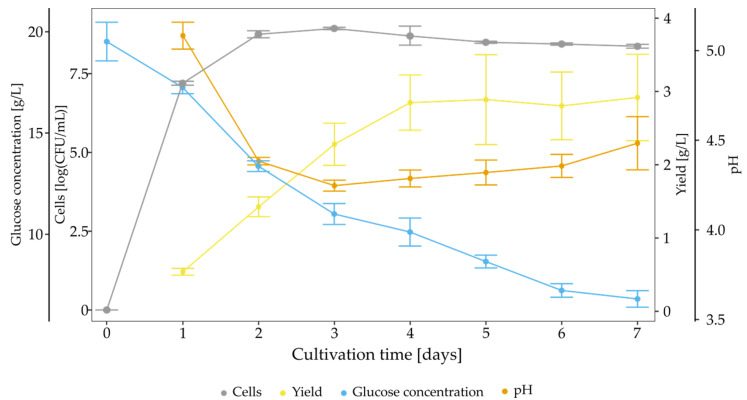
Time course of *K. hansenii* SI1 growth in SH medium.

The growth profile of *K. hansenii* SI1 growing on SH medium over seven days was examined. The yield of BNC, glucose concentration, amount of viable cells and pH were assessed each day (Figure 3). Bacterial cells grew rapidly until the second day, and afterwards, a stationary phase was observed. The maximum value of cells number reached 8.93 logCFU/mL on the third day. BNC synthesis started from the first day and linearly increased to the fourth day, where the plateau was reached. Glucose was consumed during the whole cultivation time, and the final concentration was 6.80 g/L. Thus, the medium was still rich in carbon source after culture, but cellulose production was diminished after the fourth day. The pH value decreased rapidly to 4.25 during the first three days, and subsequent, the pH value slowly increased to the final pH of 4.48. Changes in pH value can be ascribed to the fluctuations of gluconic acid concentration (Supplementary Figure S1), which is produced until the third day (2.99 g/L) and next consumed (final concentration at 2.31 g/L).

The FTIR-ATR study was conducted to confirm that a pellicle is composed of cellulose. The FTIR spectrum of a biomaterial produced by *K. hansenii* SI1 is presented in Figure 2e. The absorption bands are a fingerprint that can confirm the structure (as cellulose) and slightly vary between cellulose from different origins [41]. Two main peaks can be observed, namely at 3297 cm^−1^ and in the range of 1200–1000 cm^−1^. The first one corresponds to the –OH stretching. At the same time, a series of bands are assigned to the stretching of C-O-C of sugar rings and C-O stretching vibrations of the primary (C6) and the secondary hydroxyl (C2, C3) groups [42,43]. A peak at 2918 cm^−1^ is related to stretching vibrations of C-H groups, and 1652 cm^−1^ indicates deformational vibrations of –OH groups originated from bound water [44]. The absorption band at 891 cm^−1^ is assigned to the β-glucosidic linkage [45]. The FTIR spectrum does not differ from the reported spectra of BNC produced by other bacteria of the *Komagataeibacter* genus. According to Fuller et al. we do not observe impurities such as protein, lipids, nucleic acids or bacterial cells [46]. Thus, we conclude that *K. hansenii* SI1 produces chemically pure cellulose in standard culture conditions.

Although cellulose produced by *K. hansenii* SI1 does not differ chemically from other reported BNCs, we noticed that, in this case, the pellicle has an unexpectedly high ability to stretch. It could be stretched in all directions and maintain the formed shape. What is more, the pellicle can be easily manually shaped without using excessive tension. The BNC yield was not as high as other reported strains [47], but the newly isolated strain produces BNC with unique properties. These properties may find future application in medicine as, e.g., a shapable and transparent dressing (Supplementary Figure S2) or as a scaffold for three-dimensional cultures in tissue engineering by enabling shape and dimensions adjustment during culture.

### 3.3. The Impact of Culture Conditions on BNC Biosynthesis

**Figure 4:**
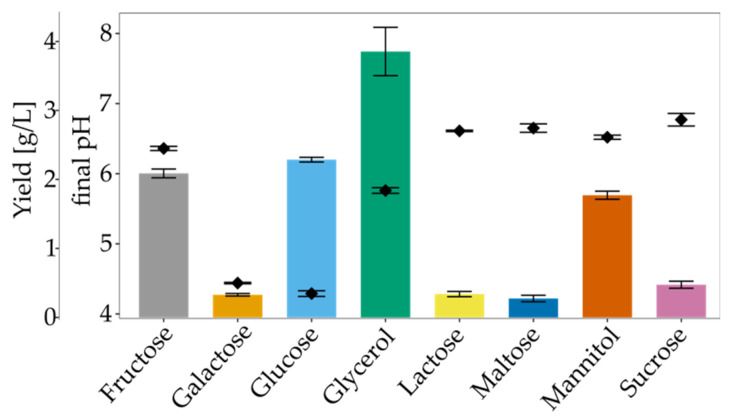
The yield of BNC and final pH in media modified with different carbon sources. Bars represent yield and points pH. Error bars represent standard deviation.

Bacteria from the *Komagataeibacter* genus can synthesise cellulose from a wide range of carbon sources, including monosaccharides, disaccharides, oligosaccharides and sugar alcohols [5]. We examined various carbon substrates as a single carbon source for BNC production. Figure 4 shows the yield and final pH form SH medium modified with 2% carbon sources in static conditions after seven days of cultivation. Cellulose synthesis was observed in all tested conditions, but the highest yield (3.85 g/L) was noted in the glycerol medium. BNC yield in the fructose- and the glucose-medium were slightly lower (2.09 g/L and 2.29 g/L, respectively). The moderate yield was recorded for mannitol, which is transformed into fructose and afterwards converted into UDP-glucose [48]. For all of the studied disaccharides and galactose, low effectiveness was observed. The highest yield in the glycerol medium may be ascribed to the altered and more efficient glycerol metabolism compared to glucose [49,50]. In this way, cellulose is synthesised without gluconic acid generation, which could explain higher BNC biosynthesis from glycerol-medium than SH medium. The high yield of BNC production in the glycerol-medium was shown for other strains, e.g., *Acetobacter* sp. V6 [49], *Ga. xylinus* CGMCC2955 [51], *K. xylinus* E26 [28] and *Gluconacetobacter* sp. RKY5 [52]. On the other hand, Abdelhady et al. who used *K. saccharivorans* PE5 and *A. xylinum* ATCC 10245, did not report satisfactory yield results from glycerol-medium in comparison to other carbon sources, e.g., mannitol, starch or sucrose [53]. Glucose and fructose gave similar results for BNC yield, although in the glucose medium, gluconic acid was generated, and the final pH was significantly lower. By taking into account pH changes during culturing, it can be seen that in the glucose medium, pH did not drop below pH 4.2. It is considered that cellulose is produced efficiently in the pH range of 4.0–7.0 [54]. Sucrose as a single carbon source was not suitable for *K. hansenii* SI1. Similar observations were reported for *K. xylinus* E25, *K. xylinus* E26, *K. hansenii* ATCC 53,582 [28] and *G. hansenii* ATCC 23,769 [55]. Mikkelsen et al. observed that cellulose production starts later (after 84 h) when sucrose is used as a carbon source [5]. On the other hand, Mohammadkazemi et al. [56] and Santos et al. [57] reported a relatively high yield of BNC in sucrose medium. It has been suggested that sucrose, which consists of glucose and fructose, is not transported through the cell membrane, but has to be previously hydrolysed into monosugars [58]. Thus, the yield of BNC in this condition is affected by the strain’s ability to the production of β-fructofuranosidase. Maltose appeared to be the least suitable carbon source for *K. hansenii* SI1, followed by galactose and lactose. Our data are in accordance with the yields obtained by Hungund and Gupta [59], Castro et al. [60], Wang et al. [50] and Rani and Appaiah [61]. Among reported strains, the most suitable carbon sources are glucose, fructose, glycerol and mannitol [25,62]. Therefore, our findings are in line with the published data.

**Figure 5:**
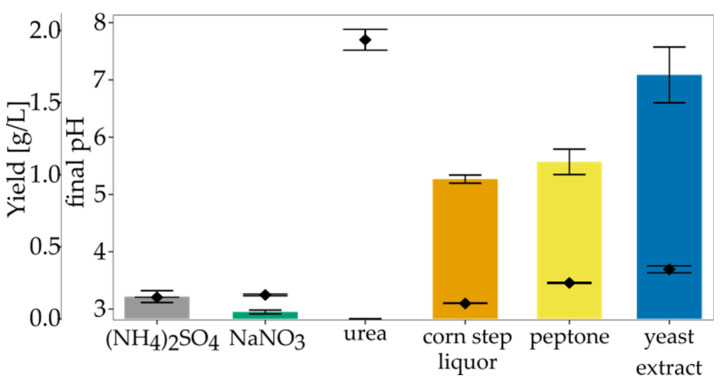
The yield of BNC and final pH in media modified with different nitrogen sources. Bars represent yield and points pH. Error bars represent standard deviation.

Nitrogen sources do not contribute directly to BNC synthesis, but they are vital for bacterial growth and survival [48]. Thus, proper nitrogen supplementation indirectly affects BNC yield. We investigated the impact of various organic and inorganic nitrogen sources of BNC synthesis in SH medium. Standard nitrogen sources (yeast extract and peptone) were substituted with another, added individually to SH medium with the concentration of 5 g/L. It was considered that organic nitrogen sources gave higher BNC yields than inorganic nitrogen sources [63]. As shown in Figure 5, medium containing yeast extract was the most suitable for BNC production, followed by medium containing peptone (1.69 g/L and 1.09 g/L, respectively). Yeast extract is rich in vitamins, especially B complex, amino acids, and trace elements that stimulate bacteria’s growth [64]. Although some authors reported the stimulatory effect of CSL on BNC yield [65,66,67], we observed a decrease in the yield compared to other organic nitrogen sources. This difference can be explained by different culture conditions, such as carbon source, additional components of medium like lactate, methionine [67]. Ramana et al. observed that the most optimal nitrogen source varies for different carbon sources [68]. In sucrose-medium, the highest BNC yield was noted for casein hydrolysate, while in the case of glucose medium, the most suitable were sodium glutamate and (NH_4_)_2_SO_4_. El-Saied et al. reported that medium containing CSL and treated molasses maximises BNC production [67]. The authors also studied the impact of CSL concentration on the BNC yield, and the optimal concentration was found to be 8%. Jang and Jeong observed a similar relationship for *G. persimmonis* KJ145 in apple juice-medium supplemented with ethanol [69].

In these conditions, we found 5 g/L to be a more economical concentration of CSL. In our study, we used 0.5% of each carbon source, and it is possible that the concentration of CSL in glucose-medium was too low to achieve a satisfying yield. Although our data showed the superiority of organic nitrogen sources, the yield was significantly lower than for standard SH medium containing both yeast extract and peptone. Our finding is supported by a study conducted for *A. xylinum* ATCC 10,245 [53]. The authors also reported that yeast extract gave the highest yield as a single nitrogen source. Still, BNC was produced the most efficiently in the presence of complex nitrogen sources (yeast extract combined with peptone or with tryptone). Santos et al. noticed that *G*. *sucrofermentas* CECT 7291 produces BNC with the highest yield only in the presence of yeast extract and peptone, or yeast extract and CSL added together [57]. *Komagataeibacter* strains require nutrients for growth and BNC biosynthesis [49]. Thus, it can be concluded that a complex, organic nitrogen source is also obligatory for efficient BNC biosynthesis by *K. hansenii* SI1.

**Figure 6:**
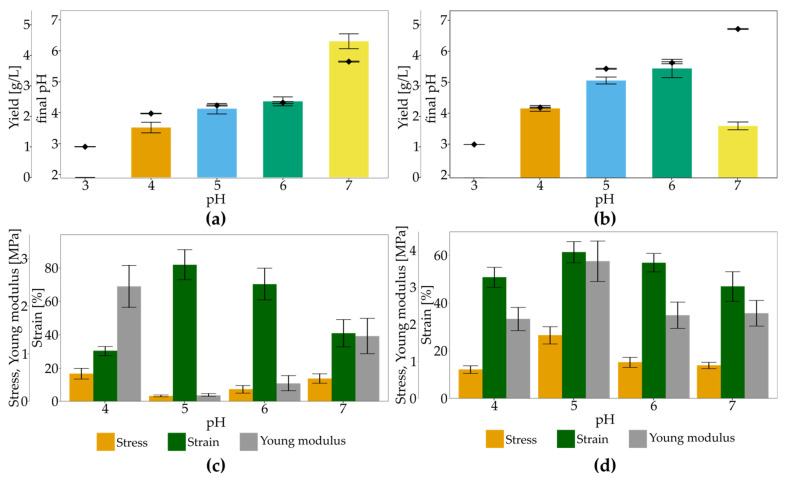
The yield (bars) of BNC and final pH (diamonds) in (**a**) SH medium and (**b**) SH medium modified glycerol at different initial pH, and mechanical properties of BNC membranes produced in (**c**) SH medium and (**d**) SH medium modified glycerol at different initial pH; error bars represent standard deviation.

The pH of the culture medium is one of the crucial parameters in terms of optimisation of BNC yield. The pH value determines the activity of enzymes correlated with bacteria growth and BNC biosynthesis [70]. For this purpose, we studied the impact of pH in an SH medium containing glucose or glycerol. Glucose medium led to the production of BNC with unique mechanical properties. At the same time, glycerol-medium resulted in the highest yield in standard conditions. *K. hansenii* SI1 was cultured for its ability to produce cellulose over the range of initial pH from 3 to 7. Above pH 7 and below pH 3, we did not observe the growth of bacteria (data not shown). From Figure 6a,b, it can be seen that the highest BNC production was obtained at pH 7.0 in glucose medium (4.46 g/L), while the most suitable pH in glycerol medium was in the range of 5–6 (3.20–3.60 g/L). At pH 5.0–6.0 in the glucose medium, the yield was diminished to 2.25 g/L–2.49 g/L. The lower pH of both media resulted in a significant decrease in the yield. The difference between optimum initial pH values for the studied carbon source can be ascribed to gluconic acid generation in the glucose medium, which is not generated in the glycerol medium. The final pH glucose medium is dropping from 7.0 to 5.7. Similar final pH (5.4–5.6) was observed for glycerol medium but initial pH at 5.0–6.0. In the case of glycerol medium, the low variation between initial and final pH results in stable conditions during culture and solves pH regulation problems. Our results agree with another research reported by Thorat and Dastager [62]. The authors studied the impact of pH in glycerol medium on BNC production by *K. rhaeticus* PG2. Similar to our results, the authors noticed an optimum pH range from 5.0 to 6.0 and the lowest yield at pH 7.0. Analogous observations in glycerol medium were reported for *Acetobacter* sp. V6 [49]. Generally, it is considered that most strains produce BNC with the highest yield in the pH range of 5.0–6.0 [27,71,72]. Still, it slightly differs between strains and dependents on culture conditions. For *K. hansenii* SI1, the 50% decrease in the yield can be observed at pH 7 in glycerol medium, while in glucose medium, it was optimal initial pH. *K. xylinus* ATCC 700,178 produced BNC with a high field in the medium with pH from 4.5 to 6.2 [73] without a specific optimum point, while for *Acetobacter* sp. A9 and *K. hansenii* AS.5, the optimum was 6.5 [74] and 5.5 [75], respectively. There are also known low- and high-pH resistant strains. Castro et al. described *K. medellinensis* strain from Colombian vinegar, producing BNC efficiently in pH at 3.5 [60]. On the other hand, Pourramezan et al. [76] and Raghunathan [77] reported that *Acetobacter* sp. 4B-2 and *Acetobacter* sp. DR-1, respectively, the most suitable pH level was 7.0, similar to our findings in the glucose medium.

Mechanical strength varies between samples from media with different initial pH and carbon sources (Figure 6c,d). In the glucose medium, high elongation was observed for membranes produced in pH range 5.0–6.0 (82% and 70%, respectively). Those pellicles were characterised by the lowest stress (0.12 MPa and 0.26 MPa, respectively) and Young modulus (0.14 MPa and 0.39 MPa, respectively). For extreme values of studied initial pH in glucose medium, the strain is decreased over two times. Although the BNC yield differed significantly for media with pH 4.0 and 7.0, the stress values were similar. Membranes produced in glycerol medium were less stretchable than BNC produced in glucose medium at pH 5.0–6.0, but this parameter did not vary highly between pH variants (from 47% to 61%).

Moreover, values of stress and Young modulus were significantly higher than for BNC membranes from the glucose medium. Pellicles produced at pH 5.0 in the glycerol medium had stress and Young modulus of 1.70 MPa and 3.70 MPa, respectively, while those parameters were 14 times and 26 times lower in the case of glucose medium, respectively. The difference in tensile properties between BNC membranes produced in different carbon sources can be ascribed to the crystallisation process [51]. In glycerol medium, bacteria produce cellulose with the lowest porosity [78] and the relative amorphous regions in the structure are reduced [49,62]. During culturing without high pH variations, bacteria synthesise larger cellulose microfibrils, which results in a compact structure [51]. These conclusions are in accordance with our findings. In the case of glycerol medium, membranes were rigid and compact irrespective of the initial pH of the culture medium. Denser arrangement of fibres in BNC form glycerol medium than from glucose medium was also reported for *K. rhaeticus* PG2 [62].

On the other hand, an unstable pH environment led to BNC production with higher porosity [6], which is more susceptible to stretching. We observed the highest difference between initial and final pH in case of glucose medium. Achieved pellicles were highly stretchable and porous (Figure 2c). We conclude that BNC characteristics produced in the glycerol medium do not expand the unique mechanical properties, such as the high ability to stretch, which is not observed for other *Komagataeibacter* strains. Thus, the next experiments will focus on modifying only glucose medium with standard initial pH (5.7), although glycerol as a single carbon source results in an improved yield.

### 3.4. The Influence of Culture Additives on BNC Biosynthesis and Mechanical Properties

**Figure 7:**
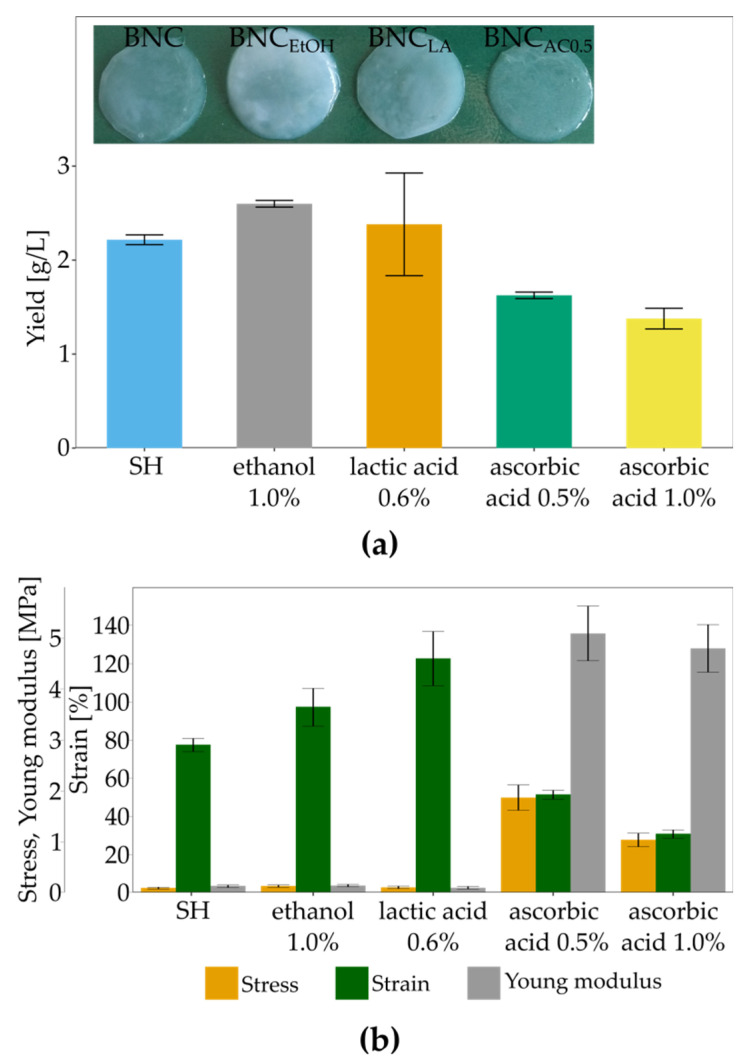
Impact of different culture enhancers on (**a**) BNC yield and (**b**) mechanical properties; error bars represent standard deviation.

Secondary substrates, such as organic acids, alcohols, vitamins and amino acids, have been proven as suitable enhancers of BNC biosynthesis [49,52,53,79,80]. Thus, we investigated the impact of ethanol, lactic acid vitamin C on BNC yield, structure and mechanical properties. Adding ethanol into the culture medium results in changes in global gene expression and the metabolic profile [81]. Ethanol also affects the enzymes from the BNC biosynthesis pathway [56]. Those changes cause an increase in BNC production. Moreover, ethanol diminishes the number of Cel (-) forms of bacteria, which do not produce bacterial cellulose [82]. On the other hand, lactate could stimulate BNC biosynthesis other than ethanol. The initial stage switches carbon flux to the TCA cycle and promotes cell growth [66]. Lactate functions as an additional energy source, generated during its oxidation into pyruvate [65]. Although vitamin C has been shown as a good enhancer for BNC biosynthesis for several *Komagataeibacter* strains [83], the direct impact of this vitamin reminds unknown. In Figure 7a, the BNC yields in the presence of culture supplements are shown. It can be seen that only ethanol improved the BNC production by *K. hansenii* SI1, but only by 18%. Reported studies show that ethanol highly increases the BNC yield. Our previous study [84] studied the effect of different ethanol concentrations on BNC production by *K. xylinus* E25. The yield was improved by 380% in the case of a 1% concentration of ethanol. Volova et al. reported a 2.2 times higher yield for *K. xylinus* B-12068 cultured in the presence of 3% of ethanol [28]. Additionally, Son et al. observed that *Acetobacter* sp. V6 in ethanol-supplemented medium produced 3.1 times more cellulose than in the medium without ethanol [64]. On the other way, El-Saied et al. noticed only a 6% improvement in the yield in the case of *Gluconacetobacter* subsp. *xylinus* ATCC 10,245 [67], which is closer to our results.

The yield in the lactic acid-supplemented medium did not differ from the yield in the SH medium. These data contradict the findings of Jang and Jeong [69] and Matsuoka et al. [66], who reported the enhanced cellulose production in lactate-medium. According to Matsuoka et al. adding 0.15% lactate into fructose-medium caused increased yield from 0.7 g/L to 3.2 g/L [66]. Jang and Jeong, who used apple juice as a carbon source, reported that at 1% concentration, lactate improved the yield 6.17-fold [69]. However, specific authors reported a lower impact of lactate on BNC biosynthesis. Jung et al. observed only a 32% increase in BNC yield in *Acetobacter* sp. V6 cultured in glycerol-medium [49]. Differences in response to the presence of lactate in the culture medium can be ascribed to varied growth rates and dynamics of BNC production by various *Komagataeibacter* strains and diverse cultivation strategies.

Although Keshk and Atykyan et al. reported stimulatory impact of vitamin C on the BNC yield in *Gluconacetobacter xylinus* (ATCC 10,245, IFO 13,693, 13,772 and 13,773) [83] and *Gluconacetobacter sucrofermentans* VKPM B-11267 [85], we observed a reverse effect for *K*. *hansenii* SI1. The higher was vitamin C concentration, the lower the amount of achieved cellulose. Other authors [86] reported data similar to our findings. They studied lower concentrations of vitamin C, namely 0.01% and 0.04%, and noticed a concentration-dependent decrease in BNC production by *K. xylinus* PTCC1734. As the mechanism of vitamin C action on *Komagataeibacter* metabolism is not yet established, the diverse response of various strains cannot be discussed in detail. Pandit et al. described the inhibitory effect of sodium ascorbate on growth rate and biofilm formation by *Bacillus subtilis* by reducing EPS production [87]. The authors concluded that vitamin C inhibits bacterial quorum sensing and other regulatory mechanisms related to biofilm formation. We observe a difference in soluble EPS (Table S1; extraction according to Fang and Catchmark [88]) between BNC from SH-medium and vitamin C supplemented medium (78 mg/L, 53 mg/L and 27 mg/L for SH medium, SH medium supplemented with 0.5% vitamin C and SH medium supplemented with 1.0% vitamin C, respectively). There were no differences in hard to extract EPS levels between culture variants (approx. 290 mg/L). One might suspect that the metabolism of vitamin C and its effects on cellulose and other EPS is similar to the mechanism described for *B. subtilis* [87]. Still, the molecular aspects of vitamin C metabolism for the genus *Komagataeibacter* require further study.

**Figure 8:**
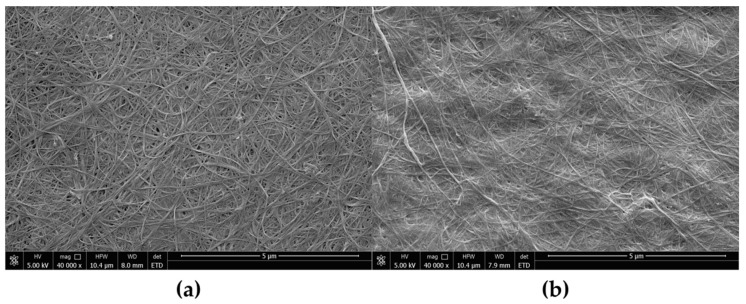
Scanning electron microscope images of BNC synthesised by *K. hansenii* SI1 in SH medium supplemented with (**a**) ethanol and (**b**) lactic acid; the images were recorded at a magnification of 40,000× (bar—5 µm).

The pellicles from ethanol- and lactic acid-supplemented media were rigid and not easily stretched. SEM images show (Figure 8) that the three-dimensional structure was changed in both cases. The fibres formed a dense structure with diminished porosity, unlike the loose arrangement in the control sample (Figure 2c). As a result, the tensile parameters of both variants were different (Figure 7b). Maximum stress at break was improved by 19.7 times in the case of BNC produced in the presence of lactic acid and by 10.9 times for BNC from ethanol-medium. The rigid structure of pellicles resulted in decreased strain from 77% to 31% and 51%, respectively. The most important change was observed for Young Modulus, 37–39 times higher than control BNC (from SH medium) due to strain and highly increased stress. Increased Young modulus for pellicles from ethanol and lactic acid supplemented media can be ascribed to the rigid structure and decreased porosity. Similar observations were reported for *K. xylinus* E25 cultured in SH medium with the addition of lactic acid, ethanol or both supplements [84]. In the case of this strain, both additives affected the structure of BNC and its mechanical properties. Membranes were compact with low porosity and were characterised by improved tensile strength (from 1.42 MPa to 4.0 MPa and 5.9 MPa for ethanol and lactic acid medium, respectively) and Young modulus (from 6.7 MPa to 20.7 MPa and 23.4 MPa for ethanol and lactic acid medium, respectively).

**Figure 9:**
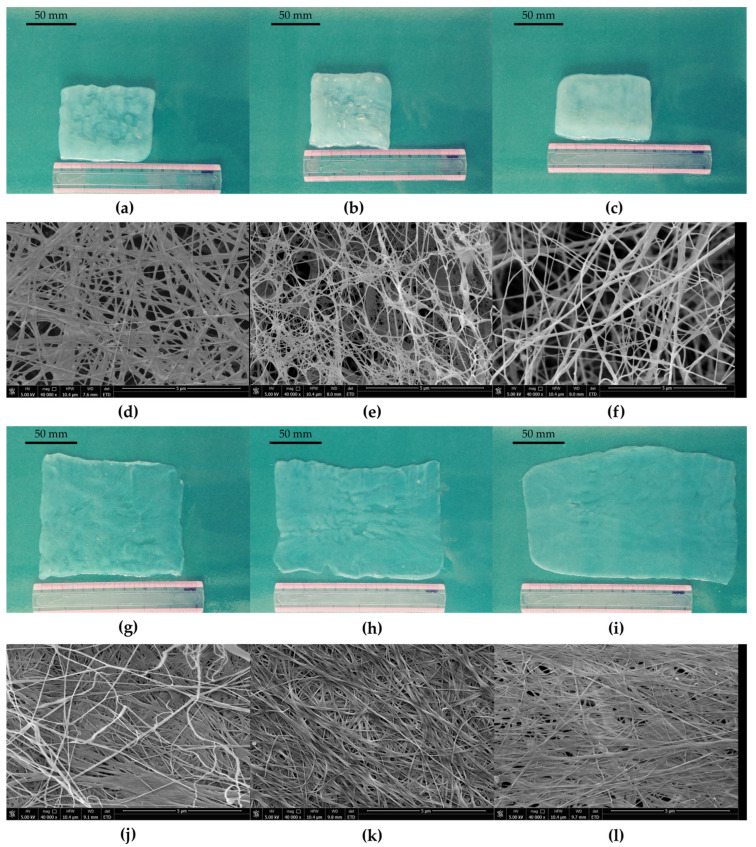
Photographs and SEM images of BNC synthesised by *K. hansenii* SI1 in SH medium (**a**,**d**) before and (**g**,**j**) after stretching; photographs and SEM images of BNC synthesised by *K. hansenii* SI1 in SH medium supplemented with 0.5% vitamin C (**b**,**e**) before and (**h**,**k**) after stretching; photographs and SEM images of BNC synthesised by *K. hansenii* SI1 in SH medium supplemented with 1.0% vitamin C (**c**,**f**) before and (**i**,**l**) after stretching; the SEM images were recorded at a magnification of 40,000× (bar—5 µm).

In contrast, vitamin C improved the porosity of membranes (Figure 9e,f) and positively affected the ability of pellicles to stretch. BNC membranes could easily manually spread on a flat surface (Supplementary Video S1). Raiszadeh-Jahromi et al. did not observe a significant difference in surface morphology caused by vitamin C, but they noticed lower compactness of cellulose layers in cross-section [86]. The authors did not mention the higher plasticity of membranes, which is observed for BNC produced by *K. hansenii* SI1. The surface of spread pellicles increased with the increasing concentration of vitamin C in the culture medium (Figure 9g,i). Fibres in the structure were orientated parallel to the direction of stretching (Figure 9k,l). The behaviour of BNC produced in vitamin C supplemented medium during hand-spreading is in accordance with mechanical properties. Both stress and Young modulus are much lower than the reported data. Costa et al. who used *G. hansenii* UCP1619, determined the Young modulus of BNC in wet state at approx. 10 MPa [89]. *K. xylinus* E25 produced membranes in SH medium with Young modulus equal to 9.0 MPa [35]. In this study, we achieved Young modulus at 130 kPa for membrane produced by *K. hansenii* SI1 in SH medium and at 142 kPa and 100 kPa from vitamin C supplemented medium (0.5% and 1%, respectively). Du et al. found that stress for BNC produced by *Ga. xylinus* isolated from Chinese persimmon vinegar was 15.2 MPa [90], similar to Kwak et al. who reported stress at 12.1 MPa for BNC produced by *Acetobacter* sp. A10 [91]. The strain (elongation) was found at 13–36% for pure, never-dried cellulose membrane [89,91,92,93]. As shown in Figure 7b, the strain of BNC membranes produced by *K. hansenii* SI1 is higher (77%). It increases with the addition of vitamin C to the culture medium (97% and 123% for 0.5% and 1% of vitamin C, respectively). To the best of our knowledge, such a high level of strain has not been yet reported.

### 3.5. The Impact of Vitamin C on BNC Biosynthesis and Chemical Structure

**Figure 10:**
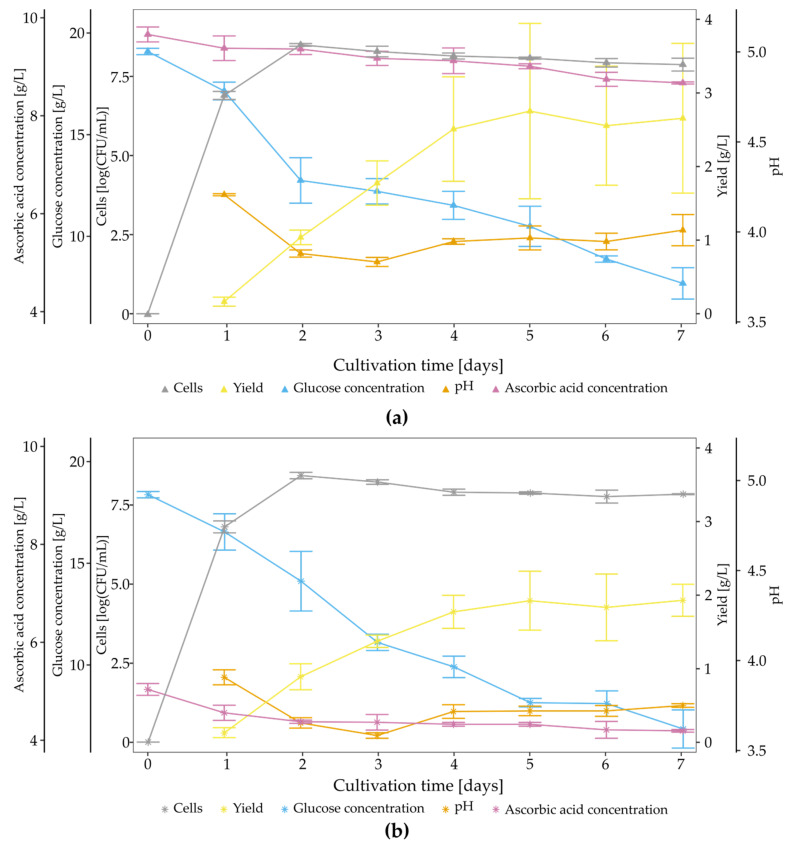
Time course of *K. hansenii* SI1 growth in (**a**) SH medium supplemented with 0.5% vitamin C and (**b**) SH medium supplemented with 1.0% vitamin C. Error bars represent standard deviation.

As stated above, *K. hansenii* SI1 produces BNC in the vitamin C-supplemented medium with a lower yield, but unique mechanical properties characterise the membrane. Thus, we evaluated the impact of vitamin C on membranes’ biosynthesis kinetic and chemical composition. In this study, bacteria were cultured in the presence of 0.5% and 1.0% vitamin C for seven days. The time course of cultures is presented in Figure 10. In comparison to SH medium (Figure 3), the growth rate of cells was diminished in the case of both modified media. Although rapid exponential growth can be observed within the first two days for all studied variants, the number of cells reached 8.50 logCFU/mL and 8.42 logCFU/mL (0.5% and 1.0% vitamin C, respectively), which is significantly lower than in the case of the control conditions. This difference could be one of the reasons for decreased BNC yield. The glucose consumption rate was almost unchanged, while the pH level was affected by adding vitamin C. In the case of supplemented media, the initial pH was 4.35 and 3.98 for vitamin C concentrations at 0.5% and 1.0%, respectively. It is worth mentioning that the BNC yield produced in 200 mL of medium containing 1% of vitamin C was lower (Figure 7a) than the yield in SH medium with the initial pH at 4.0 in analogous culture conditions (Figure 6a). During the time course study, bacteria were cultured in test tubes, which could affect the modified medium’s yield [94]. The difference between initial and final pH was also higher in modified media than in a medium with pH 4.0, e.g., ΔpH = 0.52 in case of 0.5% of vitamin C, while in case of a medium with initial pH 4.0, ΔpH was 0.01. Vitamin C was metabolised linearly during culture in 18% (for 0.5% concentration) and 13% (for 1.0% concentration). Although supplement was not fully metabolised in both cases, the yield and properties of membranes were changed in a concentration-dependent manner.

**Figure 11:**
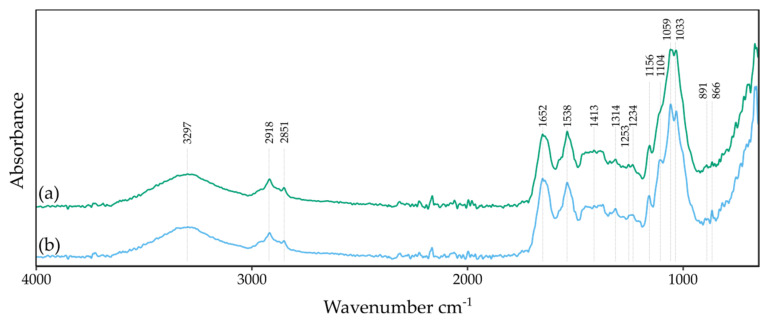
The FTIR-ATR spectra of BNC were produced in (**a**) SH medium supplemented with 0.5% vitamin C and (**b**) SH medium supplemented with 1.0% vitamin C.

The chemical composition of BNC produced in the presence of vitamin C was evaluated by FTIR and XRD analysis. The FTIR spectra presented in Figure 11 did not vary from the FTIR spectrum of the control sample (Figure 2e), except for the region between 1200 cm^−1^ and 1000 cm^−1^. With the increase of vitamin C concentration, peaks in this range are sharper and less overlapped. The bands from 1200 cm^−1^ and 1000 cm^−1^ can be ascribed to the C-O group in the primary and secondary alcohols [42,43]. Fijałkowski et al. correlated the intensity of those peaks with the progressive aggregation of cellulose microfibrils and the formation of hydrogen bonds [95]. According to the authors, differentiation of molecular structure results in enhanced crystallinity and decreased fraction of amorphous regions. Thus, based on the FTIR spectra analysis, an improved crystallinity of BNC produced in vitamin C supplemented media can be observed. This conclusion is in agreement with the XRD study.

**Figure 12:**
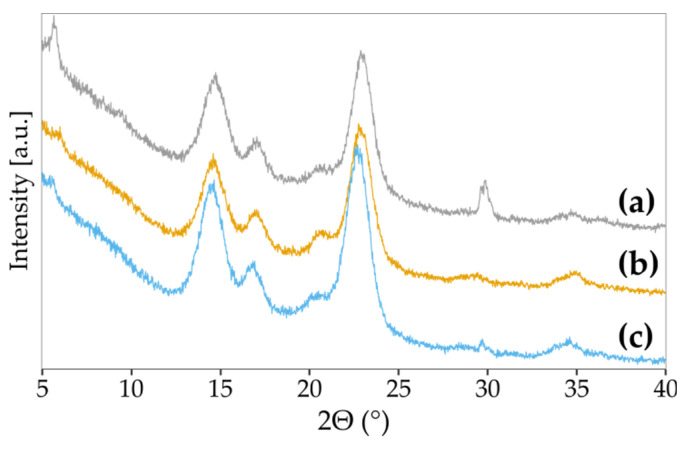
The XRD diffraction patterns of BNC were produced in (**a**) SH medium, (**b**) SH medium supplemented with 0.5% vitamin C, and (**c**) SH medium supplemented with 1.0% vitamin C.

The X-ray diffraction patterns (Figure 12) of the studied samples showed five characteristic peaks at the Bragg angle 2θ of 14.7, 17.0, 20.4, 22.7 and 34.7^o^, corresponding with the crystal planes (100), (010), (11-2), (110) and (11-4), respectively (Table 1) [96,97]. These peaks corresponded to the structure of cellulose I [38]. A higher peak intensity 2θ could easily identify the cellulose Iα at 14.7° compared to peak intensity at 17.0° [98].

The diffractograms calculated the crystallinity index (CI), the interplanar crystal distance (i.e., d-spacing), and the average crystallite size. The results are presented in Table 1. The interplanar crystal distances of the recorded peaks were similar for all BNC samples. The average crystallite size (ASC) slightly varied between the samples, but without any trend. Although the interplanar crystal distances were nearly the same, the CI of samples varied significantly. BNC produced in SH medium had the lowest crystallinity (58%), and with increasing concentration of vitamin C in the medium, CI was also increasing.

On the other hand, Keshk reported a contradictory impact of vitamin C on crystallinity for BNC produced by four different strains [83]. The author suggested that vitamin C decreases the amount of hydrogen bonds between cellulose chains, which opposes our data obtained from FTIR analysis. Nevertheless, a positive impact of vitamin C supplementation on CI was reported for *K. xylinus* PTCC 1734 cultured in the medium containing cheese whey and date syrup as a nutrient source [86]. In the studies mentioned above, it was proposed that the excess vitamin C in the culture medium influences the cellulose chains’ orientation, resulting in highly ordered structure and compact cellulose domains. The mechanism of vitamin C action was hypothesised to be the nucleation or cross-linking activity [86].

## 4. Conclusions

This study isolated a bacterial cellulose-producing strain from Kombucha and identified it as a *K. hansenii*, named SI1, based on the whole-genome sequencing. The isolated strain produces bacterial nanocellulose with unique properties such as enhanced mechanical properties, as compared to the other *Komagataeibacter* strains. To the best of our knowledge, such a high level of cellulose pellicle elongation has not yet been reported.

The production of BNC in various culture conditions was evaluated. As a single carbon source, glycerol was found to produce cellulose with the highest yield, while glucose led to the synthesis of stretchable cellulose membranes. Although glucose medium with initial pH at 7.0 stimulated high BNC production, membranes lost their unique mechanical properties. Supplementation of SH medium with common culture enhancers did not result in a significant increase in the yield.

What is the most significant, the addition of vitamin C to the culture increased porosity and improved mechanical properties of BNC, especially the strain. Moreover, those membranes were characterised by higher crystallinity than BNC produced in control conditions and were free of impurities. Based on the reported experiments, the production method of a novel type, highly stretchable bacterial cellulose was proposed. The obtained results of our study show the increased the potential of BNC in various fields, e.g., in tissue engineering as a material used for preparation of shapeable scaffold or tissue replenishment. The new type of BNC could also be a source of highly ordered cellulose nanocrystallites. The direct impact of vitamin C on *Komagataeibacer hansenii* SI1 metabolism will be a topic of a further detailed transcriptomic study which will allow us to explain changes more thoroughly in physical properties of BNC membranes.

## 5. Patents

The described method of production of BNC with enhanced porosity and high ability to stretching was subjected to an issue of Polish patent application no P.431265.

## Figures and Tables

**Table 1:** Crystallinity index, the interplanar crystal distance and the average crystallite size of BNC produced in SH medium and SH medium supplemented with 0.5% or 1.0% vitamin C.

Sample	CI [%]	Peak (101)		Peak (10-1)		Peak (012)		Peak (002)		Peak (040)	
		d_(hkl)_	ACS [nm]	d_(hkl)_	ACS [nm]	d_(hkl)_	ACS [nm]	d_(hkl)_	ACS [nm]	d_(hkl)_	ACS [nm]
SH	58	6.02	5	5.21	6	4.27	6	3.87	6	2.55	4
0.5% vitamin C	77	6.07	5	5.23	6	4.28	4	3.88	6	2.57	5
1.0% vitamin C	87	6.11	6	5.25	5	4.30	5	3.90	5	2.59	5

## Data Availability

The authors confirm that the data supporting the findings of this study are available within the article.

## Supplementary Materials

The following are available online at https://www.mdpi.com/article/10.3390/polym13244455/s1, Figure S1: Gluconic acid concentration during 7-day culture of *K. hansenii* SI1 in SH medium (grey circles), SH medium supplemented with 0.5% vitamin C (orange triangles) and SH medium supplemented with 1.0% vitamin C (blue stars). Error bars represent standard deviation. Figure S2: Hand covered with transparent stretched BNC membrane produced by *K. hansenii* SI1. Table S1: Concentration of soluble EPS and HE-EPS, Video S1: Stretching of cellulose membrane produced in the presence of 0.5% vitamin C.
