# Supplementary material for: Highly Stretchable Bacterial Cellulose Produced by Komagataeibacter hansenii SI1
Source: Polymers (Basel). 2021 Dec 19;13(24):4455. doi: 10.3390/polym13244455 (PMC8707637; doi:10.3390/polym13244455)
Supplement: Supplementary file 1 [file polymers-13-04455-s001.zip › polymers-1467921-supplementary.pdf]

Supplementary:

1. Gluconic acid concentration in time course experiment

The concentration of gluconic acid was determined each day with using K-GATE assay kit (Megazyme, Bray, Ireland) according to the manufacturer's protocol. Each test was performed at least in three replicates.

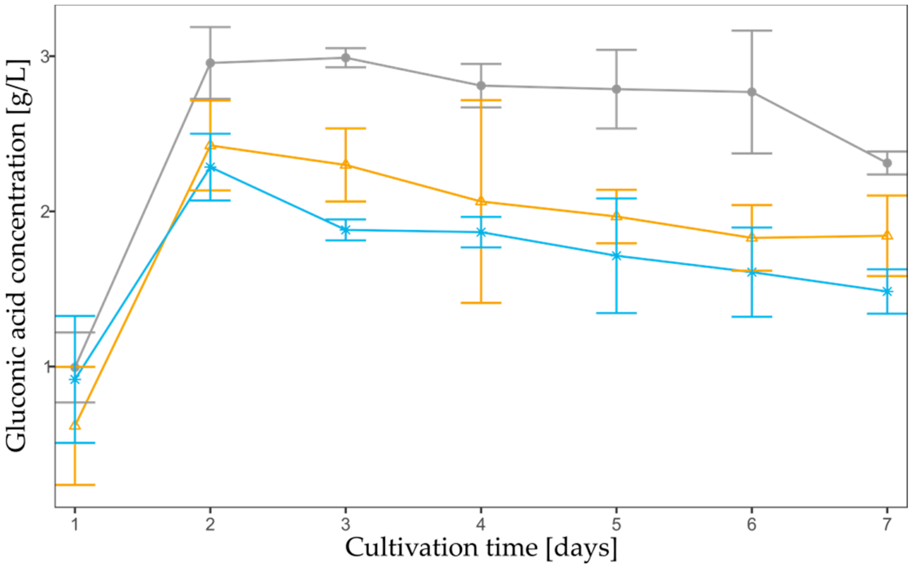

Figure S1. Gluconic acid concentration during 7-day culture of *K. hansenii* SI1 in SH medium (grey circles), SH medium supplemented with 0.5% vitamin C (orange triangles) and SH medium supplemented with 1.0% vitamin C (blue stars). Error bars represent standard deviation.

2. Transparency of stretched membranes

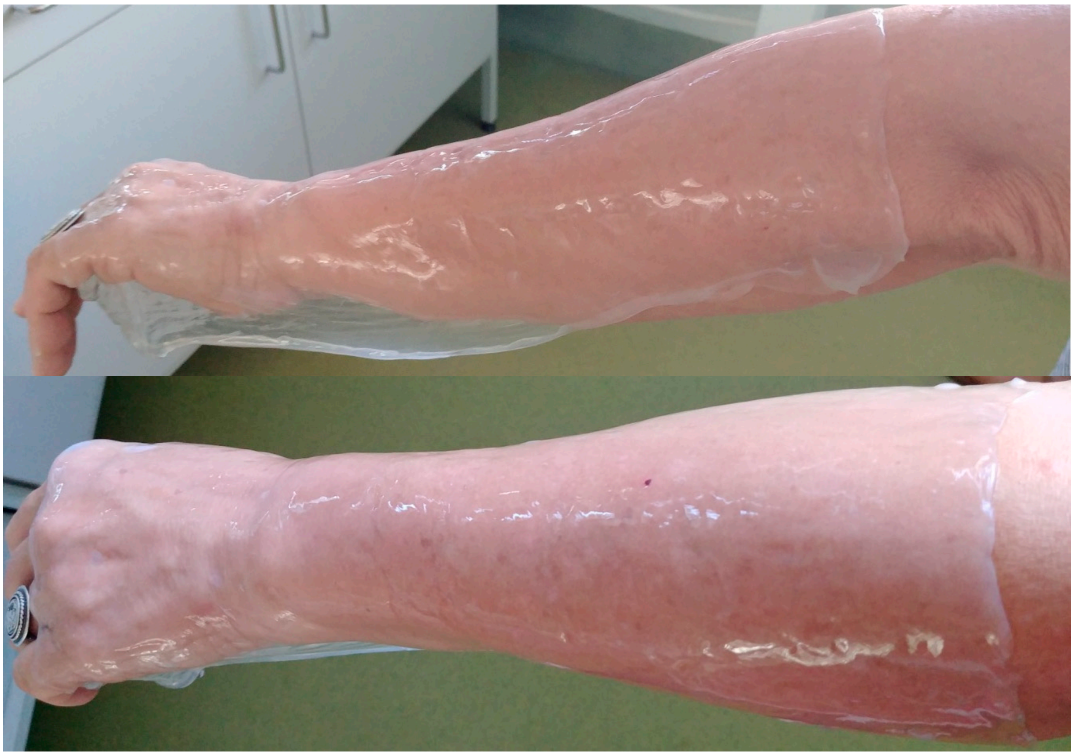

Figure S2. Hand covered with transparent stretched BNC membrane produced by *K. hansenii* SI1.

3. Impact of ascorbic acid on production of soluble EPS and hard to extract EPS

Extraction and purification of soluble (free) EPS and hard to extract (HE) EPS was carried out according to Fang and Catchmark (2015). To harvest soluble EPS, the culture broth was centrifuged and KI was added (1%) for solubilization of proteins. The soluble EPS were precipitated by adding two volumes of ethanol, incubation 24 hours at 4°C and centrifugation. The precipitate was dissolved in water and next two volumes of ethanol were added. This process was repeated three times and finally, separated precipitate was freeze dried. The HE-EPS were isolated from cellulose membranes. Wet BNC membranes were treated with 4M NaOH solution for 24 hours at ambient temperature. Next, membrane was removed and solution was neutralized with acetic acid. Afterwards, solution was dialyzed (3.5 K molecular cut off) for 24 hours at 4°C against DI water (<0.05 μS). Solution was freeze dried and weight.

Table S1. Concentration of soluble EPS and HE-EPS

Culture variant	Soluble EPS [mg/L]	HE-EPS [mg/L]
SH medium	78.00 ± 15.59	293.42 ± 9.37
SH medium + 0.5% vitamin C	52.67 ± 4.73	289.39 ± 14.27
SH medium + 1.0% vitamin C	26.67 ± 8.08	289.08 ± 5.81

Fang, L.; Catchmark, J.M. Characterization of cellulose and other exopolysaccharides produced from Gluconacetobacter strains. *Carbohydrate Polymers* **2015**, *115*, 663-669.
